# Non-invasive type 2 diabetes risk scores do not identify diabetes when the cause is β-cell failure: The Africans in America study

**DOI:** 10.3389/fpubh.2022.941086

**Published:** 2022-09-23

**Authors:** Annemarie Wentzel, Arielle C. Patterson, M. Grace Duhuze Karera, Zoe C. Waldman, Blayne R. Schenk, Christopher W. DuBose, Anne E. Sumner, Margrethe F. Horlyck-Romanovsky

**Affiliations:** ^1^Section on Ethnicity and Health, Diabetes, Endocrinology, and Obesity Branch, National Institute of Diabetes, Digestive and Kidney Diseases, National Institutes of Health, Bethesda, MD, United States; ^2^Hypertension in Africa Research Team, North-West University, Potchefstroom, South Africa; ^3^South African Medical Research Council, Unit for Hypertension and Cardiovascular Disease, North-West University, Potchefstroom, South Africa; ^4^National Institute of Minority Health and Health Disparities, National Institutes of Health, Bethesda, MD, United States; ^5^Institute of Global Health Equity Research, University of Global Health Equity, Kigali, Rwanda; ^6^Department of Health and Nutrition Sciences, Brooklyn College, City University of New York, New York, NY, United States

**Keywords:** type 2 diabetes, risk score, African (Black) diaspora, β-cell failure, insulin resistance, diabetes screening

## Abstract

**Background:**

Emerging data suggests that in sub-Saharan Africa β-cell-failure in the absence of obesity is a frequent cause of type 2 diabetes (diabetes). Traditional diabetes risk scores assume that obesity-linked insulin resistance is the primary cause of diabetes. Hence, it is unknown whether diabetes risk scores detect undiagnosed diabetes when the cause is β-cell-failure.

**Aims:**

In 528 African-born Blacks living in the United States [age 38 ± 10 (Mean ± SE); 64% male; BMI 28 ± 5 kg/m^2^] we determined the: (1) prevalence of previously undiagnosed diabetes, (2) prevalence of diabetes due to β-cell-failure vs. insulin resistance; and (3) the ability of six diabetes risk scores [Cambridge, Finnish Diabetes Risk Score (FINDRISC), Kuwaiti, Omani, Rotterdam, and SUNSET] to detect previously undiagnosed diabetes due to either β-cell-failure or insulin resistance.

**Methods:**

Diabetes was diagnosed by glucose criteria of the OGTT and/or HbA1c ≥ 6.5%. Insulin resistance was defined by the lowest quartile of the Matsuda index (≤ 2.04). Diabetes due to β-cell-failure required diagnosis of diabetes in the absence of insulin resistance. Demographics, body mass index (BMI), waist circumference, visceral adipose tissue (VAT), family medical history, smoking status, blood pressure, antihypertensive medication, and blood lipid profiles were obtained. Area under the Receiver Operator Characteristics Curve (AROC) estimated sensitivity and specificity of each continuous score. AROC criteria were: Outstanding: >0.90; Excellent: 0.80–0.89; Acceptable: 0.70–0.79; Poor: 0.50–0.69; and No Discrimination: 0.50.

**Results:**

Prevalence of diabetes was 9% (46/528). Of the diabetes cases, β-cell-failure occurred in 43% (20/46) and insulin resistance in 57% (26/46). The β-cell-failure group had lower BMI (27 ± 4 vs. 31 ± 5 kg/m^2^ *P* < 0.001), lower waist circumference (91 ± 10 vs. 101 ± 10cm *P* < 0.001) and lower VAT (119 ± 65 vs. 183 ± 63 cm^3^, *P* < 0.001). Scores had indiscriminate or poor detection of diabetes due to β-cell-failure (FINDRISC AROC = 0.49 to Cambridge AROC = 0.62). Scores showed poor to excellent detection of diabetes due to insulin resistance, (Cambridge AROC = 0.69, to Kuwaiti AROC = 0.81).

**Conclusions:**

At a prevalence of 43%, β-cell-failure accounted for nearly half of the cases of diabetes. All six diabetes risk scores failed to detect previously undiagnosed diabetes due to β-cell-failure while effectively identifying diabetes when the etiology was insulin resistance. Diabetes risk scores which correctly classify diabetes due to β-cell-failure are urgently needed.

## Introduction

Improving detection of undiagnosed type 2 diabetes is an urgent concern in sub-Saharan Africa, where 54% of diabetes is undiagnosed and prevalence is expected to increase by 143% by 2,045 (1). Compared to high income countries, many patients with type 2 diabetes living in sub-Saharan African countries are younger, do not have obesity, and present with relative β-cell-failure (2) rather than insulin resistance (2–6). Yet this phenotype is not unique in sub-Saharan Africa and has been shown in population studies from Asia [China (7), Korea (8) and India (9)], Europe [Switzerland (10)], and the Americas [Brazil (11), US (12) and Trinidad and Tobago (13)]. Indeed, a high rate of diabetes has been reported in people without obesity from low-and middle income countries (14). Current type 2 diabetes screening guidelines are based on studies in populations where insulin resistance is the primary pathogenic mechanism of type 2 diabetes.

The American Diabetes Association and International Diabetes Federation encourage the use of non-invasive risk prediction scores as affordable approaches to identifying patients at increased risk of type 2 diabetes who should be referred for diagnostic tests (1, 15, 16). Although diabetes risk scores are not applied when an individual's diagnosis is known, for individuals whose outcome is unknown, such risk scores are applied for the purpose of screening. Type 2 diabetes risk scores rely on demographic, anthropometric, and clinical risk factors that commonly appear together in at-risk individuals such as age, family history of diabetes, BMI, and blood pressure, yet none of these risk tools consider distinct type 2 diabetes phenotypes and etiologies (5, 17).

Type 2 diabetes risk scores, including the simplified Cambridge (18), simplified Finnish Diabetes Risk Score (FINDRISC) (19, 20), Kuwaiti (21), Omani (22), Rotterdam (23) and SUNSET (24) universally suppose that diabetes occurs due to obesity-linked insulin resistance since they all rely on body mass index (BMI) and/or waist circumference. Yet, data in Black people from sub-Saharan African countries suggest that β-cell-failure in the absence of obesity is a frequent cause of abnormal glucose tolerance (a term combining prediabetes and diabetes) (3, 5). Type 2 Diabetes due to β-cell-failure may often take longer to diagnose than insulin resistance (3–5). As β-cell-failure may only be diagnosed later in the course of the disease, it may in fact be a diabetes complication which triggered the screening for diabetes. Indeed, evidence from sub-Saharan African countries show that due to limited access to non-communicable disease screening, in low resource areas where access to blood testing and biomarker analyses are limited, and diagnosis of type 2 diabetes is likely to occur after the onset of clinical complications such as blindness, renal failure, and amputations (1, 3, 25). Thus, Africans with type 2 diabetes due to β-cell-failure may not be detected by current screening tools. It is unknown how well these non-invasive type 2 Diabetes risk scores perform in the detection of undiagnosed diabetes primarily due to either β-cell-failure or insulin resistance.

Therefore, the goals of this study were to determine within the Africans in America cohort the (1) prevalence of previously undiagnosed type 2 diabetes within the total cohort; (2) prevalence of type 2 diabetes due to β-cell-failure and insulin resistance; and (3) the ability of six diabetes risk scores (Cambridge, FINDRISC, Kuwaiti, Omani, Rotterdam, and SUNSET) to correctly classify patients who should be referred for diagnostic tests and detect previously undiagnosed type 2 diabetes due to either β-cell-failure or insulin resistance.

## Methods

### Population

The Africans in America cohort is a clinical protocol designed to assess cardiometabolic and psychosocial status of Black Africans born in sub-Saharan African countries and currently living in the United States (5, 26–28). Recruitment was achieved by advertisements in newspapers, flyers, social media, and posters at community events. The study was approved by the NIDDK Institutional Review Board (ClinicalTrials.gov Identifier: NCT00001853). Written informed consent was obtained.

To determine eligibility a telephone interview was conducted. To be eligible for a screening visit at the NIH Clinical Center, the prospective enrollee had to be state at their telephone interview that they were 18–65 years of age, self-identified as healthy, and that they and their parents self-identified as Black and were born in sub-Saharan Africa. In addition, they had to specifically deny a history of diabetes, liver or kidney disease, anemia, or thyroid disease. Pregnancy tests were performed in women of child-bearing age and had to be negative.

**Figure 1:**
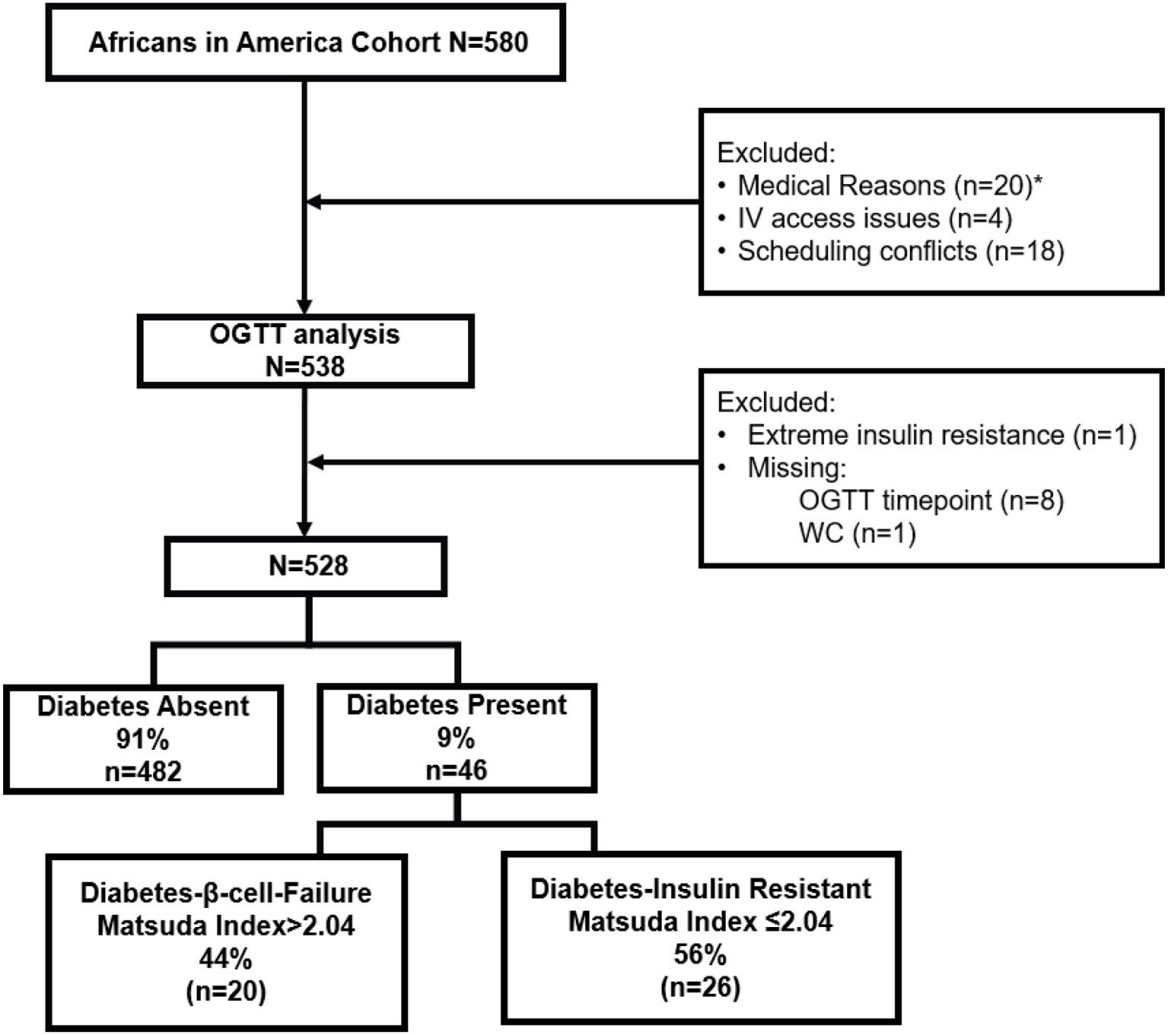
Flow diagram of study design. *Medical Reasons included anemia (*n* = 12), elevated liver transaminases (*n* = 1), declined blood draw (*n* = 3), pregnancy (*n* = 3), hypothyroidism (*n* = 1).

Five hundred and eighty African-born Black people living in metropolitan Washington, DC completed the telephone interview and proceeded to the screening visit for a medical history, physical examination, electrocardiogram, and routine blood tests (Figure 1). Questionnaires determined age of immigration, years in the U.S., family history of diabetes, use of antihypertensive medication, and smoking status.

Forty-one individuals did not proceed from the Screening Visit to the oral glucose tolerance test (OGTT). Reasons for exclusion were anemia (*n* = 12), elevated liver transaminases (*n* = 1), declined blood draw (*n* = 3), pregnancy (*n* = 3), hypothyroidism (*n* = 1), IV access issues (*n* = 4) and scheduling conflicts (*n* = 18).

Following OGTT analysis, 9 individuals were excluded. One participant had a fasting insulin concentration of 172 pmol/L (normal < 30 pmol/L) and was diagnosed with extreme insulin resistance. Eight individuals had a missing value for glucose or insulin during the multi-sampled OGTT.

One participant did not have a waist circumference value, and as waist circumference is part of the risk score calculations, was excluded from analyses.

The remaining 528 participants were included in all data analyses.

### Oral glucose tolerance test visit

After an overnight 12 h fast, participants came to the NIH Clinical Center at 7AM. Resting vitals were measured. Height, weight, waist circumference, and blood pressure (BP) were measured. Body mass index (BMI) was calculated (kg/m^2^). Obesity was defined as BMI≥30 kg/m^2^ (29). Waist circumference was measured at the superior border of the iliac crest at the end of expiration, and the mean of three values was recorded (30).

Baseline blood samples were obtained for fasting plasma glucose (FPG), insulin, and HbA1c. Post Glucola consumption (Trutol 75, Custom Laboratories) blood samples were taken at 0.5 h, 1 h, 1.5 h, and 2 h to determine glucose and insulin concentrations and calculate the AUC for glucose and insulin responses. Timepoints baseline, 0.5 h, 1 h and 2 h were used to calculate the Matsuda Index.

After the OGTT, a computerized tomographic (CT) scan (Siemens and Somatom Force Scanner) with adipose windows designed to measure visceral adipose tissue (VAT) was performed (31).

### Diabetes diagnosis

A diagnosis of diabetes required: FPG≥126 mg/dL (7.0 mmol/L) and/or 2 h glucose≥200 mg/dL (11.1 mmol/L) and/or HbA1c ≥ 6.5% (48 mmol/mol) (32).

### Insulin resistance status

Insulin resistance status was determined by Matsuda Index (5, 33):


$$\begin{array}{l} \left(\frac{10,000}{\sqrt{fasting\;glucose\;\times fasting\;insulin\;\times mean\;glucose\;\times mean\;insulin}}\right) \end{array}$$

A higher Matsuda Index denotes greater insulin sensitivity. As diagnostic category insulin sensitive was defined as Matsuda Index > 2.04, and insulin resistance was defined as the lowest quartile for our population distribution of Matsuda Index (≤ 2.04).

The oral Disposition Index, which is a measure of β-cell-function adjusted for insulin sensitivity, was calculated using the Matsuda index, as the product of insulin secretion (ISI) and insulin sensitivity (Matsuda Index). Where insulin secretion index was calculated as:


$$\begin{array}{l} ISI=\frac{AUC\;for\;insulin\;from\;0\;to\;120\;minutes}{AUC\;for\;glucose\;from\;0\;to\;120\;minutes} \end{array}$$

### Group assignment by diabetes and insulin resistance status

Participants were categorized in two ways: presence or absence of type 2 diabetes, and presence or absence of insulin resistance, resulting in two etiology groups (Figure 1) (2, 5):

**Group 1: Diabetes-β-Cell-Failure**
This group had diabetes due to β-cell-failure because diabetes occurred in the absence of insulin resistance (Matsuda > 2.04).
**Group 2: Diabetes-Insulin-Resistance**
This group had diabetes due to insulin resistance (Matsuda ≤ 2.04).

### Assays

Hemoglobin and hematocrit were measured in EDTA-anticoagulated whole blood (Sysmex XE-5000). Insulin was measured in serum and glucose, cholesterol, TG, HDL in plasma using a Roche Cobas 6000 analyzer (Roche Diagnostics, Indianapolis, Indiana). LDL was calculated using the Friedewald equation (34). HbA1c values were determined by HPLC using National Glycohemoglobin Standardization Program (NGSP)-certified instruments manufactured by BioRad Laboratories (Hercules, CA, USA).

### Diabetes scores

We included six diabetes risk scores and tested for their ability to detect previously undiagnosed type 2 diabetes in African descent populations: Cambridge, Simplified FINDRISC, Kuwaiti, Omani, Rotterdam, and SUNSET (35). Risk score components included age, sex, family history of diabetes, antihypertensive medication/hypertension, resting heart rate, BMI, waist circumference, and smoking (Table 1). Scores were applied as continuous variables and dichotomized at optimal cut-points.

**Table 1:** Components of the diabetes prevalence scores.

**Score**	**Age**	**Sex^a^**	**Family history of diabetes**	**Antihypertensive medication or hypertension^b^**	**Resting heart rate**	**BMI^a^**	**Waist circumference^a^**	**Smoking**
**Cambridge** **(****18****)** Developed in a British population	•	•	•	•		•		•
**FINDRISC** **(****20****)** Developed in a Finnish population	•		•	•		•	•	
**Kuwaiti** **(****21****)** Developed in an Arabic population	•		•	•			•	
**Omani** **(****22****)** Developed in an Arabic population	•		•	•		•	•	
**Rotterdam** **(****23****)** Developed in a Dutch population	•	•		•		•		
**SUNSET** **(****24****)** Developed in a Dutch population with European, Caribbean, South Asian, and African ancestry	•		•	•	•	•	•	

^a^For scores without sex as a variable, BMI and Waist Circumference were sex-specific.

^b^The SUNSET included both BP medication and Hypertension diagnosis by SBP/DBP.

### Statistical analysis

Unless stated otherwise, data are presented as Mean ± SE. Comparisons with the Diabetes-Absent group were adjusted for age. One-way analysis of variance (ANOVA) with Bonferroni corrections for multiple comparisons were used to compare the three groups (Diabetes-Absent, Diabetes-β-Cell-Failure and Diabetes-Insulin-Resistance) and categorical variables were compared by chi-square and the Dunn test. Unpaired *t*-tests were used to compare Diabetes-Absent and Diabetes Present groups. Area Under the curve for glucose and insulin were calculated using the trapezoidal rule.

Six risk scores were calculated for each participant and mean values compared between groups. Receiver operator characteristics (ROC) curves showed the relationship between specificity and sensitivity. Area under the receiver operator characteristic curve (AROC) assessed the ability of each risk score to detect diabetes by etiology group (*P* < 0.05). ROC was calculated for three models:

#### Model A (*n* = 528)

- n = 46 participants with Diabetes-β-Cell-Failure or Diabetes-Insulin-Resistance
- *n* = 482 participants without diabetes

#### Model B (*n* = 502)

- *n* = 20 participants with Diabetes-β-Cell-Failure
- *n* = 482 participants without diabetes

#### Model C (*n* = 508)

- *n* = 26 participants with Diabetes-Insulin-Resistance
- *n* = 482 participants without diabetes

The optimal cut-point for each risk score to detect previously undiagnosed diabetes in the total cohort (Model A) was calculated as:


$$\sqrt{\left[{\left(1-sensitivity\right)}^{2}+{\left(1-specificity\right)}^{2\;}\right]}$$

(16).

The six risk scores were dichotomized at the optimal cut point of Model A.

All statistical analyses were performed with STATA17 (College Station, Texas).

## Results

The final sample of 528 Black people born in sub-Saharan African countries was 64% male, mean age 38 ± 10y (range 20–65y), and mean BMI 28 ± 5 kg/m^2^ (range: 18.4–42.2 kg/m^2^). African regions of birth were West (50%, 266/528), Central (19%, 103/528), East (30%, 159/528). The six participants from southern African countries were grouped with the Central African countries. Most participants were male and from West Africa, similar to known immigration patterns to the United States. The prevalence of type 2 diabetes did not differ between African regions of origin, nor were there any significant differences in risk score components i.e., age, sex, BMI, waist circumference, visceral adipose tissue, FPG, 2-h glucose, HbA1c, family history of diabetes or blood pressure medication usage. Therefore, we analyzed the cohort as a single group.

### Diabetes etiology and diabetes prevalence

Prevalence of previously undiagnosed diabetes was 9% (46/528). Of the diabetes cases, β-cell-failure occurred in 43% (20/46), and insulin-resistance in 57% (26/46). The participants with Diabetes-β-Cell-Failure had been living in the U.S. longer and immigrated at a younger age compared to the Diabetes-Insulin-Resistance group (both *P* < 0.01). Mean 2 h-glucose and HbA1c did not differ between diabetes etiology groups. Yet, the Diabetes-β-Cell-Failure group had lower FPG, lower OGTT glucose levels at 0.5 h and 1 h (*P* < 0.05) than the Diabetes-Insulin-Resistance group. In addition, fasting insulin levels, and OGTT insulin at 0.5 h, 1 h, 1.5 h, and 2 h were significantly lower in the Diabetes-β-Cell-Failure group (*P* < 0.001). Compared to the Diabetes-β-Cell-Failure group, AUC for both glucose and insulin responses during the OGTT were significantly greater in the Diabetes-Insulin-Resistance group (*P* < 0.001). However, oral disposition index was significantly higher in the Diabetes-β-Cell-Failure group compared to the Diabetes-Insulin-Resistance group (*P* = 0.002) (Table 2).

**Table 2:** Population characteristics in diabetes-absent and diabetes etiology groups.

**Variable^a^**	**Total population** ***N =* 528**	**Diabetes absent** ***N =* 482**	**Diabetes β-cell-failure** ***n =* 20**	**Diabetes insulin-resistance** ***n =* 26**
Sex (% Male)	64%	64%	90%	69%
Age (years)	37 ± 10	38 ± 10	43 ± 10	45 ± 10
Age at immigration (years)	26 ± 11	26 ± 11	23 ± 8	32 ± 10^***###b^
Number of years in the U.S.	12 ± 11	12 ± 11	20 ± 9***	14 ± 10^##^
BMI (kg/m^2^)	28 ± 5	28 ± 5	27 ± 3	33 ± 4^**###^
Waist circumference (cm)	91 ± 12	90 ± 11	91 ± 7	107 ± 7^***###^
Waist circumference (cm), Women	90 ± 13	89 ± 12	85 ± 6	115 ± 6^***###^
Waist circumference (cm), Men	91 ± 11	90 ± 12	92 ± 7	102 ± 8^***##^
Visceral adipose tissue (cm^2^)	100 ± 69	97 ± 68	119 ± 65**	183 ± 63^***###^
Obesity	29%	27%	10%**	77%^***###^
Blood pressure medication (%)	8%	7%	10%	23%
Family history of diabetes (%)	28%	28%	35%	27%
Smoking status (%)	5%	5%	25%***	0%^**###^
HbA1c (%)	5.4 ± 0.7	5.4 ± 0.7	6.1 ± 0.9***	6.6 ± 1.5***
Fasting plasma glucose (mg/dL)	92 ± 13	90 ± 14	108 ± 21***	126 ± 35^***#^
Glucose at 0.5 h (ng/dL)	139 ± 27	136 ± 23	165 ± 32**	192 ± 49^**#^
Glucose at 1 h (ng/dL)	151 ± 42	145 ± 34	210 ± 39***	245 ± 56^***#^
Glucose at 1.5 h (ng/dL)	144 ± 46	134 ± 32	228 ± 30***	255 ± 62***
Glucose at 2 h (mg/dL)	133 ± 41	123 ± 26	227 ± 22***	248 ± 60***
Glucose AUC during the OGTT	544 ± 124	521 ± 88	756 ± 94**	867 ± 196^***#^
Fasting insulin (pmol/L)	7 ± 7	7 ± 5	5 ± 2*	17 ± 7^***###^
Insulin at 0.5 h (pmol/L)	77 ± 60	79 ± 62	35 ± 24**	59 ± 23^*##^
Insulin at 1 h (pmol/L)	85 ± 62	87 ± 63	45 ± 25***	86 ± 33^###^
Insulin at 1.5 h (pmol/L)	82 ± 68	81 ± 61	51 ± 24***	107 ± 55^*###^
Insulin at 2 h (pmol/L)	72 ± 56	70 ± 56	77 ± 34*	113 ± 62^***#^
Insulin AUC during the OGTT	280 ± 182	282 ± 186	183 ± 87**	308 ± 116^***###^
Matsuda index	5.6 ± 3.8	5.8 ± 3.7	5.2 ± 2.3*	1.8 ± 0.5^***###^
Oral disposition index	2.3 ± 1.0	2.5 ± 0.3	1.1 ± 0.5***	0.7 ± 0.4^***##^

^a^Data expressed as mean ± SD.

^b^Comparisons by one-way ANOVA with Bonferroni corrections for multiple comparisons, categorical variables compared by: Chi-square and Dunn Test; Comparisons to Diabetes-Absent group were adjusted for age.

^*^Comparison with diabetes-absent, ^*^P < 0.05, ^**^P < 0.01, ^***^P < 0.001.

^#^Comparison of Diabetes-β-Cell-Failure vs. diabetes-insulin-resistance, ^#^*P* < 0.05, ^##^*P* < 0.01, ^###^*P* < 0.001.

**Figure 2:**
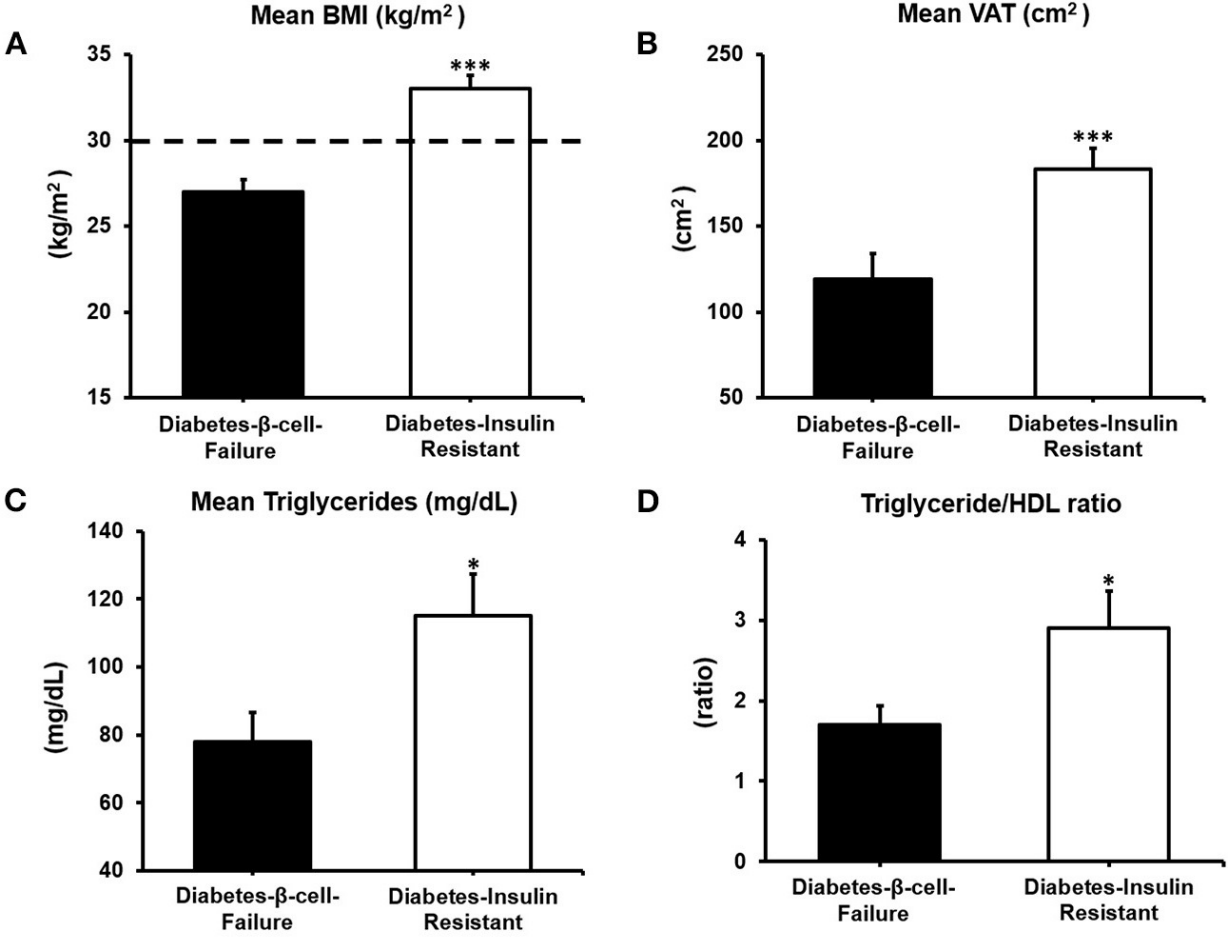
Body composition and lipid parameters according to etiology of diabetes: **(A)** Body Mass Index (BMI). **(B)** Visceral Adipose Tissue (VAT). **(C)** Triglycerides and **(D)** Triglyceride/HDL ratio. ^***^P < 0.001; ^**^P < 0.01; *P < 0.05.

The Diabetes-β-Cell-Failure group had lower BMI, lower waist circumference and lower VAT compared to the Diabetes-Insulin-Resistance group (all *P* < 0.001) (Figure 2). Obesity prevalence was also lower in Diabetes-β-Cell-Failure vs. Diabetes-Insulin-Resistance (10% vs. 77%) (*P* < 0.001). TG levels were lower in the Diabetes-β-Cell-Failure group (*P* < 0.05), but no significant differences were noted for TC, LDL, and HDL levels between etiology groups.

### Risk score comparison

**Figure 3:**
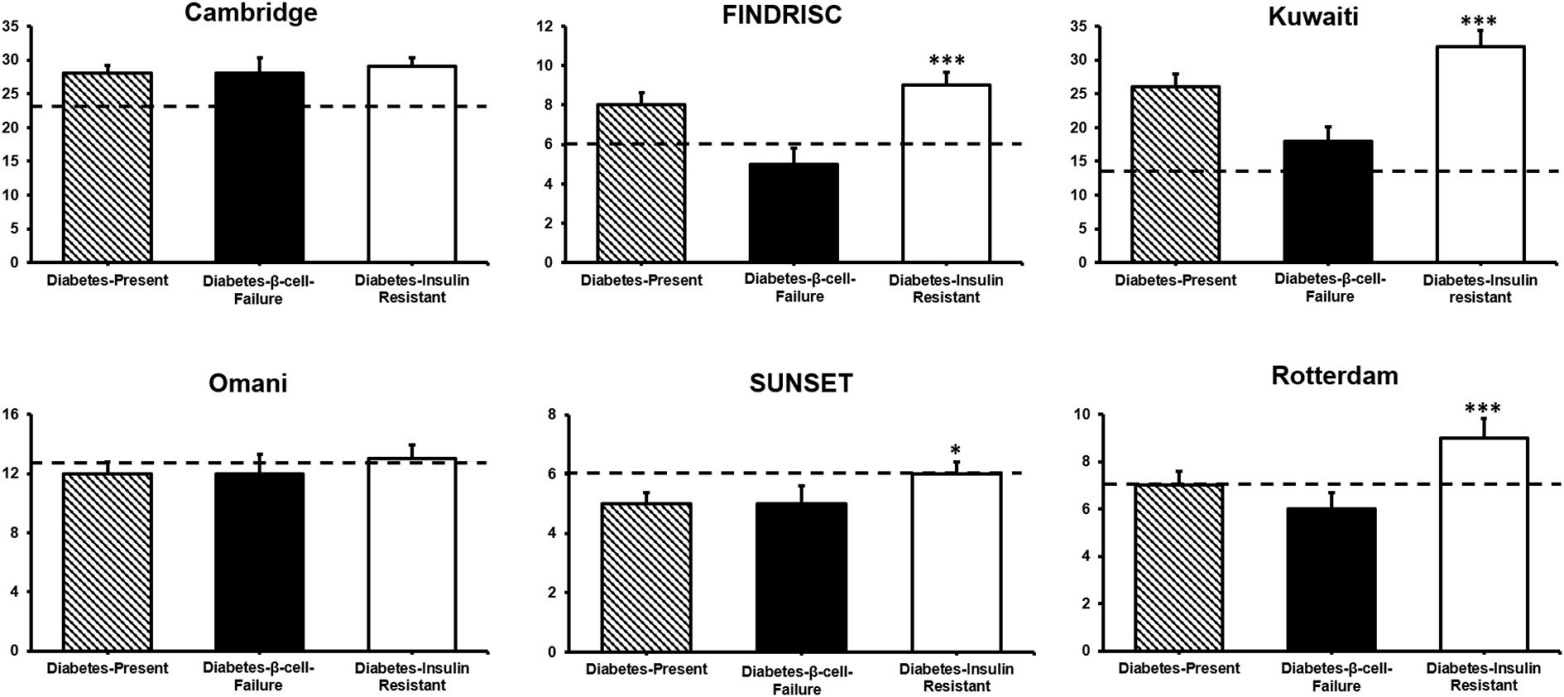
Diabetes risk scores by diabetes etiology. Comparison between diabetes β-Cell-Failure and diabetes-insulin-resistance ***P < 0.001; *P < 0.05. Dotted line shows the respective cut-point of each score predicting diabetes in this cohort.

The Diabetes-β-Cell-Failure group showed significantly lower scores for FINDRISC, Kuwaiti, Rotterdam, and SUNSET than the Diabetes-Insulin-Resistance group (all *P* < 0.05). However, there were no significant differences for the Cambridge and Omani scores between Diabetes-β-Cell-Failure and Diabetes-Insulin-Resistance groups (Figure 3; Supplementary Table 3).

Table 3 shows AROC for the Cambridge, FINDRISC, Kuwaiti, Omani, Rotterdam, and SUNSET in the Model A (Diabetes-Absent + Total Diabetes), Model B (Diabetes-Absent + Diabetes-β-Cell-Failure), and Model C (Diabetes-Absent + Diabetes-Insulin-Resistance). All scores showed indiscriminate to poor predictability in Diabetes-β-Cell-Failure. Scores had poor to excellent predictability for Diabetes-Insulin-Resistance.

**Table 3:** Area under the receiver operator characteristic curve for predicting type 2 diabetes.

**Risk score**	**Model A** **predicting total diabetes** ***AROC***	**Model B** **predicting diabetes β-Cell-Failure *AROC***	**Model C** **predicting diabetes insulin-resistance *AROC***
Cambridge risk score^a^	0.68***	0.62*	0.69***
FINDRISC, simplified	0.64***	0.49	0.73***
Kuwaiti score	0.72***	0.57	0.81***
Omani score	0.65***	0.59	0.67***
Rotterdam score	0.66***	0.55	0.73***
SUNSET score^b^	0.65***	0.55	0.70***

Model A (n = 528): Includes 46 participants with Diabetes-β-Cell-Failure OR Diabetes-Insulin-Resistance and 482 participants without diabetes.

Model B (n = 502): Includes 20 participants with Diabetes-β-Cell-Failure and 482 participants without diabetes.

Model C (n = 508): Includes 26 participants with Diabetes-Insulin-Resistance and 482 participants without diabetes.

^a^Cambridge was modified: calculated without steroidal Rx.

^b^SUNSET was modified: did not calculate the risk based on ethnicity score, nor included family History of CVD.

Bold values indicate statistically significant results. ***P < 0.001; **P < 0.01; *P < 0.05.

**Figure 4:**
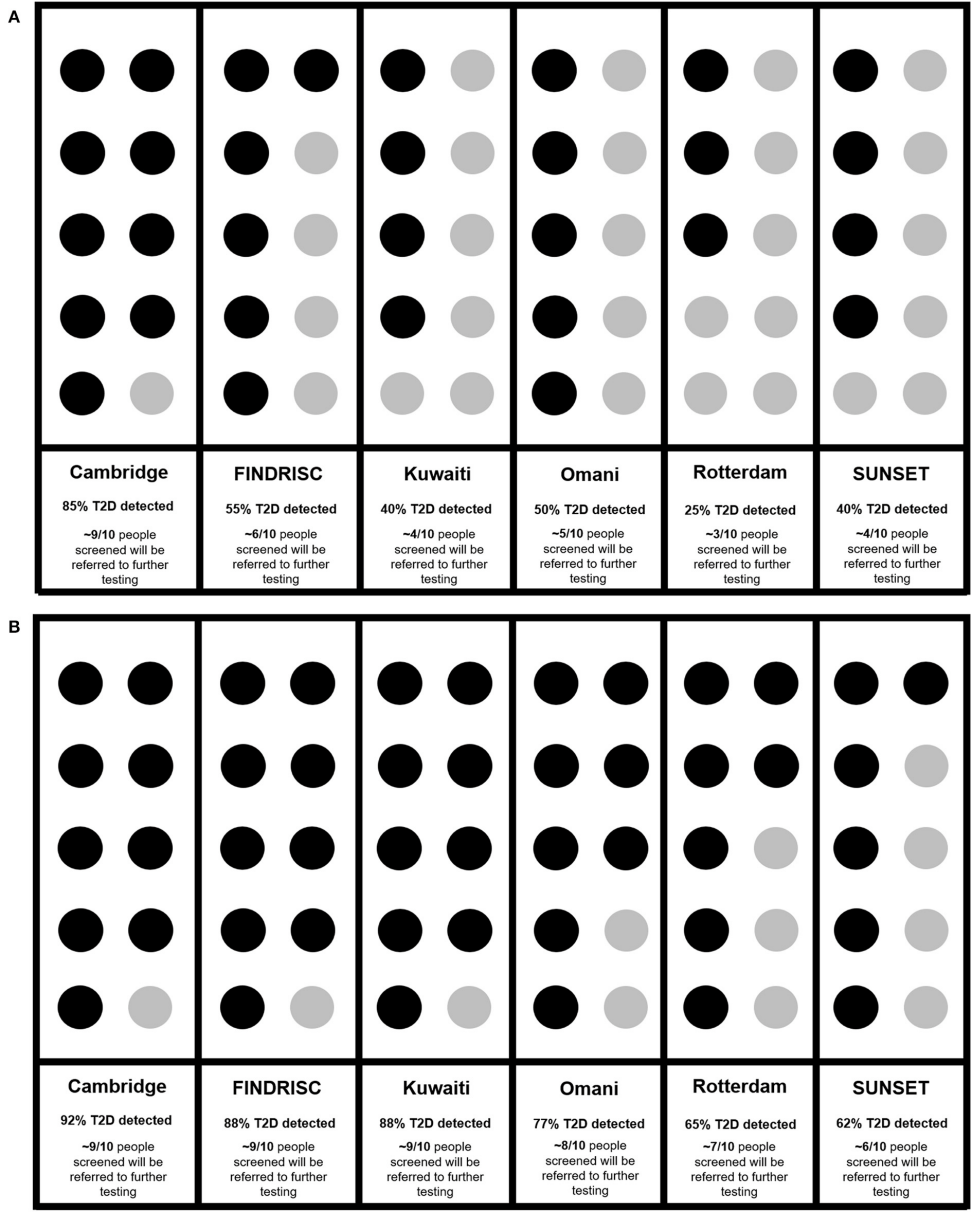
**(A)** Ability of risk scores to identify diabetes in every 10 diabetes cases due to β -Cell-Failure. **(B)** Ability of risk scores to identify diabetes in every 10 diabetes cases due to insulin resistance. Cambridge (18), FINDRISC (20), Kuwaiti (21), Omani (22), Rotterdam (23), SUNSET (24).

When examining the ability of each risk score to correctly identify ten participants with Diabetes-β-Cell-Failure, Cambridge correctly detected 9/10 (85%), FINDRISC detected 6/10 (55%), Kuwaiti detected 4/10 (40%), Omani detected 5/10 (50%), Rotterdam detected 3/10 (25%), and SUNSET detected 4/10 (40%). In contrast, when predicting Diabetes-Insulin-Resistance, Cambridge detected 9/10 (92%), FINDRISC detected 9/10 (88%), Kuwaiti detected 9/10 (88%), Omani detected 8/10 (77%), Rotterdam detected 7/10 (65%) and SUNSET detected 6/10 (62%) (Figures 4A,B).

## Discussion

Non-invasive diabetes risk scores failed to detect previously undiagnosed diabetes in Africans when the etiology was β-cell-Failure, but they effectively detected diabetes due to insulin resistance. When diabetes was due to β-cell-Failure, Cambridge was somewhat effective. Yet, this should be interpreted with caution, as Cambridge also produces a high rate of false positives and therefore might divert sparse resources to unnecessarily refer more people for diagnostic diabetes testing.

### Risk of diabetes-β-cell-failure without obesity

Even when validated in African-descent populations in the United Kingdom (36), South Africa (15), The Netherlands (24) and United States (16), non-invasive diabetes risk scores do not accurately identify diabetes due to β-cell-failure. In the current study, the only risk score that shows promise in detecting diabetes due to β-cell-failure, is the Cambridge score. Cambridge was able to identify 88% of all diabetes-cases screened. As Cambridge scores multiple levels of BMI, not just the presence or absence of obesity, it might retain relative accuracy (18). The Cambridge score may therefore be the most useful, specifically in populations where β-cell-failure is suspected to be the primary cause of diabetes. As diabetes due to β-cell-failure predominantly occurs in the absence of obesity, screening programs are unlikely to identify these as “high-risk” cases. Such risk-underestimation means that diabetes diagnosis occurs only after the onset of clinical complications. Nevertheless, as the modified Cambridge score was able to accurately identify previously undiagnosed diabetes due to both insulin resistance and β-cell-failure, this score appears to perform relatively well regardless of diabetes etiology. However, this must be interpreted with caution, as low specificity indicates that Cambridge over-estimates risk. Therefore, applying Cambridge in low resource settings, would divert scarce resources to unnecessary diagnostic testing of people who do not have diabetes.

### Obesity-linked risk in the diabetes-insulin-resistance group

The Diabetes-Insulin-Resistance etiology group did indeed show phenotypic characteristics associated with insulin resistance: higher BMI, higher waist circumference and higher visceral adipose tissue in addition to higher triglyceride/HDL ratio. This supports the notion that non-invasive risk scores assume that the primary cause of diabetes is obesity-linked insulin resistance. The six risk scores showed better discriminatory ability to identify undiagnosed diabetes due to insulin resistance compared to β-cell-failure. In three population studies, specifically FINDRISC was linked to insulin resistance (16, 37, 38), and shown to be effective in detecting diabetes in patients with obesity. A cross-sectional validation study of the Cambridge and Rotterdam scores in an Irish population, (39) reported that anthropometric components (BMI and waist circumference) of the risk scores demonstrated the greatest discriminatory ability and largest contribution to the overall score. Our findings support this, as all six scores effectively detect diabetes in the insulin resistant group, the group that also had the highest prevalence of obesity. Body mass index and/or waist circumference are two of the strongest predictors of risk in all six scores. The value of waist circumference as a proxy for VAT and predictor of insulin resistance is illustrated by the significantly higher VAT, in the Diabetes-Insulin-Resistance group vs. the Diabetes-β-Cell-Failure group as previously shown (5, 40).

Although the six non-invasive diabetes risk scores remain valuable for the detection of undiagnosed diabetes primarily caused by insulin resistance, risk scores relying on BMI and waist circumference fail to predict diabetes due to β-cell-failure, as only 10% (*n* = 2) had obesity. This is of major concern, as Black people born in sub-Saharan African countries may be at greater risk of developing diabetes at a lean-to-normal BMI and have β-cell-failure or secretory dysfunction, rather than peripheral insulin resistance (3–6, 41, 42).

### Family history

Family history of diabetes has been shown to be a strong, independent predictor of diabetes risk (43, 44). Yet, in populations originating from low- and middle-income countries, family history might be underreported because family medical history is unknown, or family members live or have lived with undisclosed diabetes (43). Although, in our population, family history of diabetes did not significantly differ between diabetes etiology groups, we recognize that family history could be a powerful predictor of diabetes risk.

### Measuring insulin resistance not degree of glycemia

Adding HOMA-IR and HOMA-β did not increase FINDRISC's discriminatory accuracy (45, 46). Our findings show that insulin resistance status plays a key role in all scores, except Cambridge. This is also supported by findings from the San Antonio Heart study showing that by adding HOMA-IR or HOMA-β to the predictive model the discriminatory capability of the FINDRISC model significantly improves (47). We found that not only FINDRISC, but also Rotterdam, Kuwaiti, Omani, and SUNSET's detection abilities were greatly influenced by insulin resistance status.

Furthermore, markers of glycemia, fasting plasma glucose, 2-h glucose and HbA1c did not differ between diabetes etiology groups. However, the oral disposition index differed significantly. Compared to the Diabetes-β-Cell-Failure group, the Diabetes-Insulin-Resistance group had considerable insulin resistance and also a lower oral disposition index. This might be explained by the lower insulin concentrations in the Diabetes-β-Cell-Failure group compared to the Diabetes-Absent group. In addition, the Diabetes-β-Cell-Failure group had minimal insulin resistance (Matsuda > 2.04). This observation of higher oral disposition index in β-cell-failure is supported by observations in members of the Luo and Kamba tribes in Kenya (48). This indicates that these non-invasive diabetes scores predict diabetes due to degree of insulin resistance, and not based on the level of hyperglycemia. This trend is supported by other studies (16, 37), where particularly FINDRISC effectively detected diabetes due to insulin resistance in populations at risk of diabetes. Tracking with insulin resistance and not the degree of glycemia make these scores inherently biased toward diabetes due to insulin resistance.

### Future directions

Participants in our study that were diagnosed with type 2 diabetes, were newly diagnosed by OGTT and thus previously undiagnosed. According to the IDF (1), 56% of people living with type 2 diabetes in Africa are undiagnosed—and our study showed that the current, non-invasive equations applied, do not detect them. Thus, patients are not receiving the benefit of early intervention to prevent possible type 2 diabetes related complications. Therefore, we advocate for modifying and optimizing these non-invasive diabetes risk scores for Africans, to identify existing diabetes.

Using Cambridge, FINDRISC, Kuwaiti, Omani, Rotterdam, and SUNSET as initial screening tools, is justified only when diabetes etiology is insulin resistance. The only diabetes risk score that may show some promise irrespective of diabetes etiology, is the Cambridge score. Previous findings from our lab suggest that a single invasive marker such as random or fasting plasma glucose rather than a battery of non-invasive variables would ensure more accurate detection of diabetes regardless of etiology. However, diabetes screening in sub-Saharan Africa is particularly challenging as diabetes screening and diagnostic tools such as random glucose, fasting plasma glucose, HbA1c, and the gold standard oral glucose tolerance test (OGTT) are either costly, invasive, inconvenient or do not perform optimally in African-born populations. A prior study from the Africans in America cohort showed that fasting plasma glucose ≥100 mg/dL alone was equivalent to the nine-variable ARIC + HbA1c prediction equation (49) despite the inclusion of BMI, age, Triglycerides, LDL, HDL and HbA1c. However, relying on plasma glucose may present logistical challenges in low- and middle-income settings where test strips and glucometers may not always be available, calibrated, or functional.

Risk scores which do not require invasive blood tests and rely on commonly collected clinical information are easy to use as screening tools in low resource and community settings. However, as diabetes due to β-cell-failure was not identified by five of the six risk scores, future risk tools for diabetes screening must account for this etiology. Early detection of diabetes due to β-cell-failure is crucial as these patients may only be identified after the onset of clinical complications such as retinopathy, renal failure, diabetic ulcers, and amputations. Furthermore, risk scores with high sensitivity and low specificity may unnecessarily increase referrals for costly diagnostic testing. Refining existing risk scores or creating new scores that either distinguish between body size categories more accurately, or do not only rely on characteristics associated with insulin resistance is required to more accurately detect diabetes due to β-cell-failure.

### Strengths and limitations

The strengths of this study are fourfold. First, this study provides glucose and insulin levels from the OGTT. This allowed us to measure estimate insulin resistance by both Matsuda index and HOMA-IR to define diabetes etiologies. These tests differentiated between and categorized diabetes etiology groups similarly, which supports the use of HOMA-IR in low resource settings as it relies on a single fasting blood sample. Second, we demonstrated that non-invasive diabetes risk scores perform well when insulin resistance is the cause of diabetes, and that score performance was comparable to other studies in the sub-Saharan African region (20).

Third, the use of CT scans to obtain accurate estimation of VAT also supported the higher BMI and waist circumference as correlates of insulin resistance. Fourth, all socio-behavioral questionnaires, and risk score variables were administered, measured, and scored by research staff, and clinical variables were measured by clinical staff which increases completeness and reliability of collected data.

The study has two main limitations. The study was cross sectional and therefore identifies current and not future diabetes risk, although, these scores have been applied to detect diabetes incidence (15). Additionally, this is a convenience sample representing Black immigrants from sub-Saharan countries living in the Washington DC metropolitan area who chose to participate in the study. Nevertheless, the characteristics of our study population are consistent with known demographics of sub-Saharan African immigrants in the US, including a majority being male and 50% originating from West Africa (46). Furthermore, our findings are similar to previous studies of newly diagnosed diabetes among Sub-Saharan Africans living in Canada (6%) (50) and urban Sub-Saharan African populations(8.4%) (51). Nevertheless, we demonstrated that current diabetes prediction equations fail when β-cell-failure is the cause yet perform well when insulin resistance is the cause of diabetes.

As a limitation, we did not have access to islet-cell antibody testing to rule out latent autoimmune diabetes in adults (LADA) (52) or genetic tests to rule out maturity-onset diabetes of the young (MODY) (53). However, mean age for newly diagnosed diabetes was 43y in the β-Cell-Failure group and 45y in the Insulin-Resistance group, thus MODY is unlikely. In addition, although participants with β-cell failure had below normal insulin secretion, insulin secretion was still present, and no study participants reported a current or past history of diabetic ketoacidosis. Therefore, LADA was also unlikely to be the cause of diabetes in these participants.

## Conclusion

At a prevalence of 43%, β-cell-failure accounted for nearly half of the cases of diabetes. All six diabetes risk scores failed to detect previously undiagnosed diabetes due to β-cell-failure while effectively identifying diabetes when the etiology was insulin resistance. Diabetes risk scores which correctly classify diabetes due to β-cell-failure are urgently needed.

## Data availability statement

The raw data supporting the conclusions of this article will be made available by the authors, without undue reservation.

## Ethics statement

The studies involving human participants were reviewed and approved by the Institutional Review Boards of the National Institutes of Health. The patients/participants provided their written informed consent to participate in this study.

## Author contributions

Conceptualization, writing—original draft preparation, and writing—review and editing: AW, AES, and MFH-R. Methodology and investigation: AW, ACP, ZCW, BRS, MGD, CWD, MFH-R, and AES. Formal analysis: AW, ACP, BRS, MFH-R, and AES. Resources: AES. Data curation: ZCW and AES. Visualization: AW, ACP, MFH-R, and AES. Supervision: AW, CWD, and AES. Project administration: AW, ACP, ZCW, BRS, CWD, MFH-R, and AES. Funding acquisition: AW and AES. All authors have read and agreed to the published version of the manuscript.

## Funding

AW was supported by the South African Medical Research Council, Unit for Hypertension and Cardiovascular Disease, North-West University, South Africa. AW, ACP, MGD, ZCW, BRS, CWD, and AES were supported by the intramural program of NIDDK. AW, MGD, and AES were also supported by the intramural program of NIMHD. AW also received support from the NIMHD William G. Coleman Minority Health and Health Disparities Research Innovation Award. MGD was also supported by Institute of Global Health Equity Research, University of Global Health Equity, Rwanda. MFH-R was supported by City University of New York, New York.

## Conflict of interest

The authors declare that the research was conducted in the absence of any commercial or financial relationships that could be construed as a potential conflict of interest.

## Publisher's note

All claims expressed in this article are solely those of the authors and do not necessarily represent those of their affiliated organizations, or those of the publisher, the editors and the reviewers. Any product that may be evaluated in this article, or claim that may be made by its manufacturer, is not guaranteed or endorsed by the publisher.
