# Supplementary material for: Non-invasive type 2 diabetes risk scores do not identify diabetes when the cause is β-cell failure: The Africans in America study
Source: Front Public Health. 2022 Sep 23;10:941086. doi: 10.3389/fpubh.2022.941086 (PMC9537602; doi:10.3389/fpubh.2022.941086)
Supplement: Supplementary file 1 [file Data_Sheet_1.docx]

**SUPPLEMENTARY Table 1: Total Population Characteristics Groups with Diabetes Present and Diabetes Absent**

| **Variable^1^** | **Total**  **N=528** | **Diabetes Absent**  **n=482** | **Diabetes Present**  **n=46** | ***P*-value^2^** |
| --- | --- | --- | --- | --- |
| **Sex (% Male)** | **64%** | **64%** | **78%** | **0.035** |
| **Age (years)** | **37±10** | **38±10** | **44±10** | **<0.001** |
| **BMI (kg/m^2^)** | **28±5** | **28±5** | **30±5** | **<0.001** |
| **Waist Circumference (cm)** | **89±11** | **90±11** | **100±11** | **<0.001** |
| **Visceral Adipose Tissue (cm^2^)** | **100±69** | **97±68** | **156±70** | **<0.001** |
| **Obesity** | **29%** | **27%** | **48%** | **0.001** |
| **Blood Pressure Medication (%)** | **8%** | **7%** | **17%** | **0.002** |
| **Family History of Diabetes (%)** | 28% | 28% | 30% | 0.704 |
| **Smoking status (%)** | **5%** | **5%** | **11%** | **0.051** |
| **HbA1c (%)** | **5.4±0.7** | **5.4±0.7** | **6.4±1.3** | **<0.001** |
| **Fasting Plasma Glucose (mg/dL)** | **92±13** | **92±14** | **116±32** | **<0.001** |
| **Glucose at 2h (mg/dL)** | **133±41** | **133±42** | **230±53** | **<0.001** |
| **Glucose AUC during the OGTT** | **544±120** | **521±87** | **800±176** | **<0.001** |
| **Fasting Insulin (pmol/L)** | **8±7** | **7±7** | **12±8** | **<0.001** |
| **Insulin at 2h (pmol/L)** | **72±58** | **71±55** | **102±86** | **<0.001** |
| **Matsuda Index** | **5.7±3.8** | **5.5±3.7** | **3.3±2.3** | **<0.001** |
| **Oral Disposition Index** | **2.3±1.0** | **2.4±0.9** | **1.0±0.5** | **<0.001** |

**^1^Data expressed as mean ± SD**

**^2^Comparisons for continuous variables were by unpaired t-test and for categorical variables by Chi-square**

**SUPPLEMENTARY Table 2: Risk Score Comparison between Groups with Diabetes Absent and Diabetes Present**

| **Risk Score^1^** | **Diabetes Absent**  **N=482** | **Diabetes Present**  **N=46** | ***P*-value** |
| --- | --- | --- | --- |
| **Cambridge** | **24±6** | **28±8** | **<0.001** |
| **FINDRISC, Simplified** | **5±4** | **8±4** | **<0.001** |
| **Kuwaiti** | **5±7** | **26±13** | **<0.001** |
| **Omani** | **9±6** | **12±5** | **<0.001** |
| **Rotterdam** | **5±3** | **7±4** | **<0.001** |
| **SUNSET** | **4±3** | **5±3** | **<0.001** |

**^1^Data expressed as mean ± SD**

**^2^Comparisons for continuous variables were by unpaired t-test**

**SUPPLEMENTARY Table 3: Risk Score Comparison According to Etiology of Diabetes**

| **Risk Score^1^** | **Diabetes**  **β-Cell-Failure**  **n=20** | **Diabetes**  **Insulin-Resistance**  **n=26** | ***P*-value^2^** |
| --- | --- | --- | --- |
| **Cambridge** | 28±10 | 29±7 | 0.826 |
| **FINDRISC, Simplified** | **5±4** | **9±3** | **<0.001** |
| **Kuwaiti** | **18±11** | **32±11** | **<0.001** |
| **Omani** | 12±6 | 13±5 | 0.377 |
| **Rotterdam** | **6±3** | **9±4** | **0.007** |
| **SUNSET** | **5±3** | **6±2** | **0.026** |

**^1^Data expressed as mean ± SD**

**^2^Comparisons by unpaired t-test**
